# Children Consider Procedures, Outcomes, and Emotions When Judging the Fairness of Inequality

**DOI:** 10.3389/fpsyg.2022.815901

**Published:** 2022-03-03

**Authors:** Lucy M. Stowe, Rebecca Peretz-Lange, Peter R. Blake

**Affiliations:** ^1^Department of Cognitive Sciences, University of California, Irvine, Irvine, CA, United States; ^2^Department of Psychology, State University of New York, Purchase, NY, United States; ^3^Department of Psychological and Brain Sciences, Boston University, Boston, MA, United States

**Keywords:** fairness judgments, procedures, distributive justice, emotions, development

## Abstract

Children tend to view equal resource distributions as more fair than unequal ones, but will sometimes view even unequal distributions as fair. However, less is known about how children form judgments about inequality when different procedures are used. In the present study, we investigated children’s consideration of procedures (i.e., resource-distributing processes), outcomes (i.e., the distributions themselves), and emotions (i.e., the emotional reactions of those receiving the resources) when judging the fairness of unequal resource distributions. Participants (*N* = 130, 3- to 8-year-olds) were introduced to a Fair Coin (different color on each side) and an Unfair Coin (same color on both sides). In two between-subjects conditions, they watched a researcher flip either the Fair or Unfair Coin in order to distribute resources unequally between two child recipients. Participants then rated the fairness of this event, provided verbal justifications for their ratings (coded for references to procedures and/or outcomes), and rated the emotional state of each recipient (from which an Emotion Difference Score was computed). Results revealed that participants rated the event as more fair in the Fair Coin than the Unfair Coin condition. References to the outcome in children’s justifications predicted lower fairness ratings, while references to the procedure only predicted lower ratings in the Unfair Coin condition. Greater Emotion Difference Scores predicted lower fairness ratings, and this effect increased with age. Together, these results show that children consider procedures, outcomes, and emotions when judging the fairness of inequality. Moreover, results suggest age-related increases in consideration of recipients’ emotions makes inequality seem less fair, even when fair procedures are used. Implications for the development of fairness are discussed.

## Introduction

*For everyone agrees that what is just in distribution must be according to worth in some sense. But they do not all mean the same sort of worth: for democrats it is freedom, for supporters of oligarchy it is wealth, for others it is noble birth, and for aristocrats it is virtue.*

*– Aristotle, Nicomachean Ethics*

As Aristotle famously noted in the Nicomachean Ethics, different groups have different moral norms concerning which procedures for distributing resources are considered fair. Children recognize several possible approaches to distributing resources, as described in classic research on the development of distributive justice (Piaget, 1932; Damon, 1980). Recent studies have expanded this work. Many studies have found that preschool children generally view equal distributions as more fair than unequal ones (Smith et al., 2013; Blake et al., 2014; Rakoczy et al., 2016; McAuliffe et al., 2017). Critically, however, children also believe that even unequal resource distributions can be fair if they are based on merit (Baumard et al., 2012; Rizzo et al., 2020), need (Paulus, 2014; Rizzo et al., 2016), rectifying past inequalities (Rizzo and Killen, 2020), shared group membership (Rhodes et al., 2018), or even a close relationship (Olson and Spelke, 2008; Paulus and Moore, 2014). In all of these cases, children view the resource distributions as fair despite being unequal, demonstrating great flexibility in recognizing different “sorts of worth” in different contexts.

We aimed to build upon this research by investigating how children evaluate the fairness of unequal resource distributions when the procedures used to create them are ostensibly fair or unfair. Recent events have highlighted how unequal distributions are sometimes the output of procedures that might still be considered fair, such as distributing vaccines to high-risk populations before low-risk ones. People’s judgments of these scenarios have important consequences, and these judgments have their roots in early childhood. The present study investigates two understudied factors which may contribute to the development of fairness judgments of unequal resource distributions: (1) children’s consideration of procedures used to distribute resources vs. the distributions themselves, and (2) children’s evaluations of the emotions of the recipients.

### Fairness Judgments Based on Procedures vs. Outcomes

Much research has examined how children evaluate distributions (i.e., “outcomes”) as fair or unfair. By at least 12 months of age, infants expect agents to create equal outcomes and prefer these agents over ones who create unequal outcomes (Geraci and Surian, 2011; Schmidt and Sommerville, 2011; Sloane et al., 2012; Ziv and Sommerville, 2017; Buyukozer Dawkins et al., 2019). Some studies even suggest that children’s experience with resource-based interactions contributes to their expectation of equal outcomes (Ziv and Sommerville, 2017). Infants also show some expectations of procedural fairness: by 20 months of age, infants expect agents to be impartial when helping others (Surian and Margoni, 2020) and by 21 months of age they expect agents to use merit to distribute resources (Sloane et al., 2012).

By the preschool years, children show both preferences and expectations for equal outcomes, judging these to be more fair than unequal outcomes and even protesting unequal outcomes (Smith et al., 2013; Rakoczy et al., 2016; Rizzo and Killen, 2016; McAuliffe et al., 2017). However, to date, less is known about children’s judgments of distributional processes (i.e., procedures) as fair or unfair, and the existing evidence of children’s commitments to procedural fairness is mixed. On the one hand, children will choose fair over unfair procedures for distributing resources (Shaw and Olson, 2014; Dunham et al., 2018), will spontaneously change a game’s unfair rules to be more equitable (Grocke et al., 2015), and will even sacrifice some resources to punish someone who distributes resources unequally (McAuliffe et al., 2015). On the other hand, preschoolers will sometimes accept and perpetrate unfair reasons for inequality (e.g., giving more to whomever started with more, Hussak and Cimpian, 2015; giving more to those who “just want more,” Schmidt et al., 2016) and will avoid fair procedures to receive an advantage (Shaw et al., 2014; Dunham et al., 2018). Thus, open questions remain about children’s reasoning about procedural fairness. Scenarios in which ostensibly fair procedures produce unequal distributions are particularly useful in revealing children’s reasoning, as these scenarios require children to weigh outcomes and procedures against each other directly.

It is important to gain clarity on how children reason about procedural fairness, as this is a major component of mature reasoning about inequality. When children first encounter social inequality in early childhood, they construct an understanding of inequalities which then informs their social attitudes, judgments, and behaviors (Rutland et al., 2010; Killen et al., 2018; Elenbaas et al., 2020). Critically, young children tend to ignore the procedures, systems, and structures that produce social inequalities. Instead, children intuitively assume that inequalities are produced by differences in groups’ intrinsic merit or inborn abilities (Hussak and Cimpian, 2015; Dunlea and Heiphetz, 2020, 2021; Peretz-Lange and Muentener, 2021), though this tendency to overlook the structures producing inequality declines over development (Vasilyeva et al., 2018; Peretz-Lange et al., 2021). We build on this research by investigating children’s nascent understanding of procedures producing inequalities.

### Fairness Judgments Based on Emotions

A second factor that may influence children’s fairness judgments is how children understand the emotional impact on the recipients of inequality. Prior work with 3-year-old participants has found that emotional reactions to unfairness may be a developmental precursor to more explicit moral judgments of unfairness (LoBue et al., 2011), and some philosophers have argued that emotions may play a theoretically central role in moral judgment (Prinz, 2006, though other philosophers argue for an alternative, rationalist view of morality, see Peacocke, 2004 and May, 2021 for reviews). Yet, little is known about how children’s developing understanding of emotions shapes their fairness judgments.

Several lines of research suggest that children’s ideas about recipients’ emotions may increasingly shape their moral judgments over development. First, classic work on the “happy victimizer effect” shows that children increasingly consider the emotions of the victims of moral transgressions with age (Nunner-Winkler and Sodian, 1988; Arsenio and Kramer, 1992; Keller et al., 2003). Research has also found that parent–child conversations while watching a television episode involve more perspective-taking and emotion-related language (e.g., “how did that make him feel?”) as children grow older, which corresponded with shifts in moral judgments (Cingel and Krcmar, 2019). Finally, recent work by Smetana and Ball (2018, 2019) also shows that children judge some moral violations more harshly than others (e.g., judging physical harm as worse than unequal resource distributions), and that these judgments corresponded with judgments of victims’ negative emotions.

In the present study, we investigated how children use emotions to inform their judgments about inequalities that are produced by either fair or unfair procedures. After children were taught about these inequalities, they were asked to rate the emotional state of the individuals who were advantaged or disadvantaged by the inequality.

### The Present Study

In the present study, we showed participants either a fair or unfair procedure producing an unequal outcome, between-subjects. We chose a large inequality (one vs. six stickers) as a strong test of whether children would view a fair procedure as outweighing the outcome. Participants were asked to rate whether this event was good or bad overall. Next, participants provided justifications for their rating, which we coded as referring to the outcome or the procedure. Finally, participants were asked to rate the emotions of the individuals who were advantaged or disadvantaged by the inequality. These diverse measures provided a rich and in-depth picture of children’s reasoning.

We predicted that (1) participants would rate the event as worse after viewing an unfair procedure compared to a fair procedure, following past work, (2) that this difference between conditions would increase with age, following evidence that children increasingly attend to the structures producing inequality with age, (3a) that references to the outcomes in the justifications would predict lower ratings of the event overall, (3b) that references to the procedure would predict lower ratings only in the unfair condition, and (4) that participants would rate the disadvantaged child as being less happy than advantaged child, and that larger differences between these two ratings would predict lower fairness ratings.

## Materials and Methods

### Participants

Children between 3 and 8 years of age (*N* = 130; 75 females; range = 36.6–107.7 months; *M* = 75.7 months; SD = 17.8) were recruited through a family database at a university lab, at a local museum, and in public parks. By age group, the sample consisted of 31 3–4 years olds (*M* = 52.7 months, SD = 6.6, 19 females), 53 5–6 years olds (*M* = 72.2 months, SD = 7.1, 27 females), and 46 7–8 years olds (*M* = 95.2 months, SD = 7.6, 29 females). An additional seventeen children were excluded from the final sample due to failing comprehension checks (14), a cognitive diagnosis revealed by the parent (2) and ending the task voluntarily (1). Demographic information was obtained on a voluntary basis and only 36% of participants provided any information. Of that subset, however, 79% were White and the average income was in the range of $100,000 to $150,000.

We used the most similar prior research (Shaw and Olson, 2014) to target a sample size of 120 children: *N* = 20 children per age group (3) per condition (2). We used G*Power to determine that a sample of 120 participants would be able to detect a medium effect size (Cohen’s *f*^2^ = 0.15; Cohen, 1992) at 95% power for a regression model with three predictors [Age (continuous), Condition and Age × Condition; Hypotheses 1 and 2] with alpha set to 0.05. We continued data collection to *N* = 130 in an effort to test more 3- to 4-year olds. Data collection ceased due to the pandemic. A *post hoc* power analysis also showed that a sample size of 130 would be sufficient to detect medium to small effects (Cohen’s *f*^2^ = 0.11) in a regression model with five predictors at a power level of 80%. Parental consent was obtained for all participants, and we also confirmed verbally with children that they wanted to participate. All procedures were approved by the university IRB.

### Procedure

The procedure consisted of a familiarization phase and a test phase. In the familiarization phase, participants were introduced to the Fair and Unfair Coins, and their comprehension was confirmed. In the test phase, participants were told about how one coin (either the Fair or Unfair Coin, depending on condition assignment) was used to distribute stickers between two other children. They then provided fairness ratings, justifications for these ratings, and emotion ratings.

#### Familiarization Phase

Participants were first shown an image of a slide and two characters (gender-matched to the participant). They were told that both characters wanted to go down the slide, but that only one could go down at a time. Participants were then told that they needed to choose a coin to help decide who could go down first and were introduced to a Fair Coin that had blue on one side and white on the other (matching the characters) and an Unfair Coin that had white on both sides. They were shown short videos in which each coin landed twice, with the blue\white coin landing once on white and once on blue and the white coin landing twice on white. They were instructed that the color the coin landed on would determine who was allowed to go down the slide first. As a comprehension check, participants were asked which coin they would like to use, and also which character would get to go down the slide first if the coin landed on blue or white. All participants in the final sample passed both comprehension check questions. Note that 11 additional participants (10 of whom were 3- and 4-year-olds) were excluded from the sample after failing the first comprehension check question, and 3 additional children were excluded after failing the second comprehension check question (2 of whom were 3- and 4-year-olds).

#### Test Phase

Participants were introduced to a laminated, drawn figure named Maya/Michael (gender-matched to the participant). They were told that Maya/Michael wanted to give stickers to two other children who had recently helped her/him, and were then shown images of these two children (also gender-matched to the participant). The child images were selected from the Child Affective Facial Expression (CAFE) data set (LoBue, 2014; LoBue and Thrasher, 2015). We used the Maya/Michael character as an intermediary who was making the decision as opposed to having the experimenter make the decision so that children would feel more comfortable saying that the result was bad, knowing that they were not criticizing the experimenter. They were told that Maya/Michael only had two packages of stickers, one package with one sticker and another with six stickers. The stickers were presented in packages so that they could not be re-allocated. Children were told that Maya/Michael would flip a coin to decide who got which package. Participants were told that if the coin landed on the color of the box (red or black) that was underneath the photo of the child, then that child would receive the package of six stickers and the other child would receive the package of one sticker. We used an inequality of 1 vs. 6 because this would be visually impressive even to young children whose number knowledge was limited.

Participants were shown a video showing two coins, a Fair Coin, which was red on one side and black on the other, and an Unfair Coin, which had the same color (either red or black) on both sides. In the video, a hand rotated each coin to show both sides and then flipped it to demonstrate its possible outcomes as in the familiarization phase. A comprehension check confirmed that participants knew how the stickers would be distributed under either outcome; all children passed this check. Participants were then told that Maya/Michael actually only had one coin, either the Fair or Unfair Coin, which represented the condition manipulation. The experimenter then flipped the coin for Maya/Michael, revealing the outcome, and the stickers were distributed accordingly.

**FIGURE 1:**
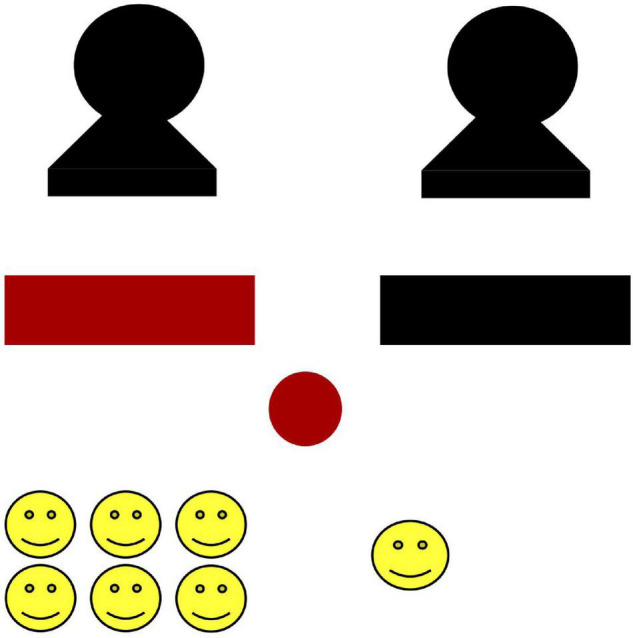
Example of the visual presentation of the result of the coin flip. Photos of real children were used in place of the silhouettes.

**FIGURE 2:**
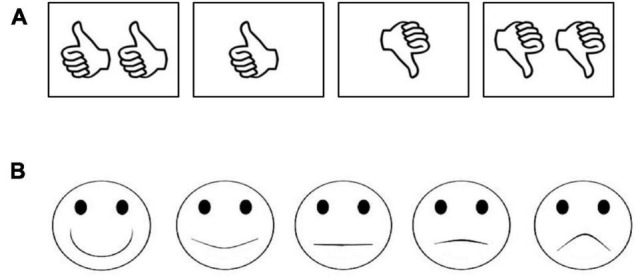
**(A)** Four-point fairness rating scale. **(B)** Five-point happy-sad scale.

Children were shown a visual presentation of the result of the coin flip (Figure 1). Participants were then asked to rate the perceived fairness of the event overall. Specifically, the experimenter said, “Maya/Michael used this coin [holding up coin] to give 1 sticker to this girl/boy and 6 stickers to this girl/boy.” This particular phrasing was used to remind participants of both the procedure (the coin) and the unequal outcome, to avoid leading participants toward relying on only the procedure or the outcome in making their judgments. The experimenter then asked, “Do you think this was really bad (1), not ok (2), ok (3), or really good (4),” pointing to the appropriate ideograph on a 4-point scale (see Figure 2A). Two versions of the scale, with either two thumbs down or up in the left position, were counterbalanced between participants. Participants were then asked why they had selected that point on the scale (e.g., “why was it really bad?”). Their responses were fully written down at the time and later coded as referring to the procedure, outcome, both, or neither.

Finally, participants were asked to predict both recipients’ emotions on a 5-point face scale (see Figure 2B). Two versions of this scale, starting with either really sad or really happy, were counterbalanced between participants. Children were asked, “how do you think this girl/boy will feel about getting 1 (or 6) sticker(s)? Will she/he feel really happy (5), a little bit happy (4), just okay (3), a little bit sad (2), or really sad (1)?” Participants were asked about the child who received more stickers and the child who received fewer, in a counterbalanced order. An Emotion Difference Score was computed as the ratings of the six-sticker child minus the ratings of the one-sticker child.

## Results

Analyses focused on evaluating our predictions that (1) participants would rate the event as worse in the Unfair Coin condition compared to the Fair Coin condition, (2) that this difference between conditions would increase with age, (3) that the more participants referred to the unfair procedure in their justifications, the worse they would rate the event, and (4) that participants would rate the child receiving six stickers as happier than the child receiving one sticker, and that larger differences between these emotion ratings would predict lower fairness ratings. All analyses were conducted in R version 4.0.2 (R Core Team, 2020) using the lm function from the lme4 package for linear regression models and the ANOVA function to compare the fit of models. For model selection, we started with a full model for each hypothesis and used the drop1 command to remove variables that did not significantly contribute to the model fit (Bolker et al., 2009).

**FIGURE 3:**
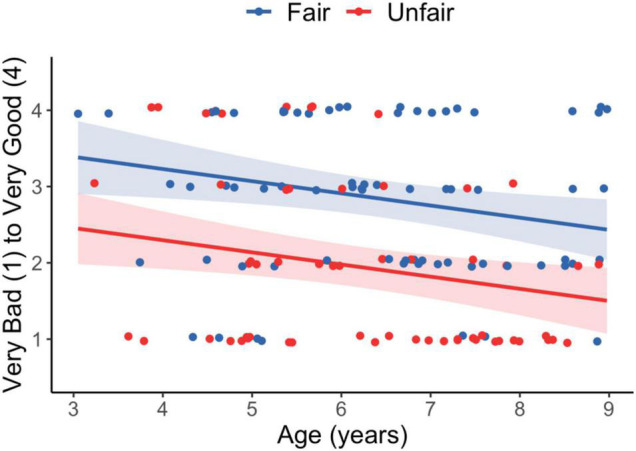
Fairness ratings as a function of Condition and Age. Shaded bands represent 95% CIs.

We first investigated whether condition impacted fairness ratings (Hypothesis 1) and whether this changed with age (Hypothesis 2). We first compared an intercept-only model to a model with Age (months), Condition (Fair, Unfair) and the interaction term. The full model significantly improved the fit to the data [*F*(3,126) = 11.42, *p* < 0.001]. To assess the need for the interaction term, we used the drop1 command (test = “F”) which suggested dropping the interaction. The main effects only model showed that children rated the task as less fair with age (*B* = −0.01, SE = 0.005, *p* < 0.05) and as less fair in the Unfair Coin condition (*B* = −0.93, SE = 0.18, *p* < 0.001). We next used the ggeffects package to obtain estimates and 95% confidence intervals for the ratings by age for each condition. This allowed us to determine whether the ratings were within the Fair or Unfair range of ratings (midpoint = 2.5). In the Fair condition, children’s ratings were in the fair range until about 8 years of age, at which point the 95% CIs included 2.5 (*B* = 2.59, 95% CI [2.28, 2.90]). In the Unfair condition, children ratings were in the fair range until about 5 years of age (*B* = 2.14, 95% CI [1.84, 2.44]). Thus, until 5 years of age, children rated the procedure plus unequal outcome as fair regardless of which coin was used (Figure 3).

Next, we investigated whether participants’ justifications of their ratings predicted their fairness ratings (Hypothesis 3). Justifications were coded by two research assistants who identified whether the explanations referred to the procedure (e.g., “because he flipped a coin”), the outcome (e.g., “because she got less”), both, or neither. The coders agreed 86.2% of the time, for a kappa of 81.3, representing near-perfect agreement. Discrepancies were resolved by the last author. Out of 123 justifications, 20% referred to only the procedure, 38% referred to only the outcome, 8% referred to both, and 34% referred to neither. Two dummy-coded binary variables were created to respectively represent whether participants did or did not refer to the procedure, and whether they did or did not refer to the outcome.

To determine whether references to the procedure or the outcome impacted children’s judgments, we created a regression model that included interactions of Procedure references (yes/no), Outcome references (yes/no) with Age and Condition. We then used the drop1 function to eliminate terms that did not significantly contribute to the model fit. The reduced model included the main effects of Age, Condition, Outcome and Procedure and the interaction of Condition × Procedure. The results showed that references to the unequal outcome predicted lower fairness ratings overall (*B* = −0.39, SE = 0.18, *p* < 0.05) and references to the procedure predicted lower fairness ratings in the Unfair Coin condition (*B* = −1.04, SE = 0.42, *p* < 0.05).

Finally, we analyzed participants’ emotion ratings as they related to their fairness ratings (Hypothesis 4). First, we sought to confirm that participants rated the child receiving six stickers as happier than the child receiving one sticker. The descriptive statistics showed the expected pattern with the recipient who received more rated close to very happy on the 5-point scale (*M* = 4.7, SD = 0.82) and the recipient who received less rated close to a little sad (*M* = 1.7, SD = 1.17). These ratings were combined into a difference score: recipient who received more minus recipient who received less. To determine the effect of the emotion difference score on fairness ratings, we created a full model that included the interaction of Emotion Difference with Age and Condition. We then used the drop1 function to eliminate terms that did not significantly contribute to the model fit. The reduced model included the main effects of Age, Condition, Emotion Difference, and the interaction of Age × Emotion Difference. The results showed that, holding Condition and Age constant, Emotion Difference scores were positively associated with fairness ratings overall (*B* = 0.61, SE = 0.28, *p* < 0.05). This effect was qualified by a significant interaction between Emotion Difference scores and Age (*B* = −0.01, SE = 0.001, *p* < 0.05), such that for older children, larger emotion difference scores predicted lower fairness ratings.

**FIGURE 4:**
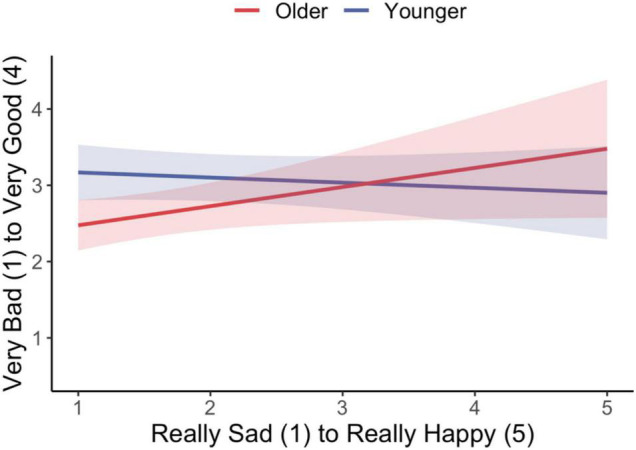
Interaction of Age and Emotion rating for the child who received less. Shaded bands represent 95% CIs.

To examine this result more closely, we ran separate models replacing the Emotion Difference score with the actual emotion ratings for the child who received less (Emotion Less) and the child who received more (Emotion More). Only the emotion ratings for the child who received less predicted fairness ratings (Figure 4). The results showed significant effects for: Age (*B* = −0.03, SE = 0.001, *p* < 0.01), Condition (*B* = −0.91, SE = 0.18, *p* < 0.001), Emotion Less (*B* = −0.58, SE = 0.29, *p* < 0.05), and the interaction of Age × Emotion Less (*B* = −0.01, SE = 0.01, *p* < 0.05). With increasing age, children who rated the child who received less as being more sad also rated the event as being less fair.

We next combined the emotion and justification ratings into a single model in order to determine whether these variables separately predicted fairness ratings. The results showed that the interactions of Condition × Procedure and Age × Emotion Less remained significant, indicating that these terms made independent contributions to children’s fairness ratings (Table 1). However, reference to Outcomes in children’s justifications was no longer significant. This change suggests that the addition of the emotion ratings explained some of the same variation as the references to outcomes in the justifications.

**TABLE 1:** Combined model including justifications and emotion ratings.

	*B* (SE)
Intercept	4.63***
	(0.67)
Age (months)	−0.03**
	(0.01)
Emotion Less	−0.54^⋅^
	(0.28)
Condition (Unfair)	−0.64**
	(0.22)
Refer to Procedure	0.72*
	(0.33)
Refer to Outcome	−0.35^⋅^
	(0.18)
Age × Emotion Less	0.01*
	(0.00)
Condition × Procedure	−1.07*
	(0.42)
*R* ^2^	0.29
Adj. *R*^2^	0.25
Number of Observations	130

***p < 0.001; **p < 0.01; *p < 0.05; ⋅p < 0.1.

## Discussion

The present study introduced child participants to either a fair or an unfair procedure which was used to distribute stickers unequally (one vs. six stickers to two child recipients). Participants were asked to rate the fairness of the event overall, justify their rating, and predict the respective emotions of the children who received one sticker and six stickers. Several key results emerged, which we discuss below, along with implications and limitations.

First, children at all ages differentiated between the two procedures, rating the event as worse in the Unfair Coin condition compared to the Fair Coin condition. Surprisingly, the fairness ratings for both conditions declined with age. By 5 years of age, children in the Unfair condition rated the event as being clearly unfair and by 8 years of age, children in the Fair condition gave average ratings that included the midpoint of the fairness scale. In fact, 50% of 7- to 8-year olds gave the event a rating of really bad or not ok in the Fair condition. These results suggest that while children attend to procedures from an early age, they place more weight on outcomes with age when evaluating how fair a distribution is. Although this result runs counter to other studies showing that procedures can override unequal outcomes with age (Shaw et al., 2014), it likely reflects children’s greater experience and stronger opinions as to how inequalities should be allocated.

Second, participants’ justifications also confirmed that attention to the procedure contributed to fairness judgments. In the Fair Coin condition, referring to the procedure predicted rating the event as more fair, but in the Unfair Coin condition, referring to the procedure predicted rating the event as less fair. Referring to the unequal outcome also predicted lower fairness ratings, as expected given the large literature showing that children view unequal outcomes as unfair. These results from participants’ justifications mirror findings from their ratings alone, although to some extent children may have been engaging in post hoc rationalization of the ratings they just gave. Future research could explore children’s reasoning in more depth by replacing the rating scale with semi-structured questions to determine what aspects of the task drew their attention the most. In sum, children integrate information about the procedure and the outcome in order to make sense of distributions as a whole, with both factors contributing to their fairness judgments.

Third, participants’ consideration of the emotions of the recipients played an increasingly important role in their fairness judgments with age. At all ages, participants rated the child who received one sticker as less happy than the child who received six stickers; however, the difference between these respective ratings increasingly predicted children’s fairness judgments with age. Specifically, results indicated that participants’ ratings of the child who received fewer resources drove these effects. However, despite this anticipation of distress for the disadvantaged recipient, children do not use this emotion information in their fairness judgments until about 5 years of age. Importantly, although the role of emotion in the generation of fairness judgments increased with age, it was not affected by the procedure used to create the inequality. Put simply, the impact of emotion on fairness judgments was driven by the outcomes.

These findings on the use of emotion information build on a rich research literature showing that infants and toddlers anticipate and respond to distress in others. This sensitivity to victims motivates prosocial actions towards the victim (Brownell et al., 2009; Svetlova et al., 2010; Dunfield, 2014; Vaish and Hepach, 2020), even when the victim does not show overt distress when harm befalls them (Vaish et al., 2009). The expectation of distress can also motivate more than just direct prosocial responses. When infants witness agents being attacked or being treated unfairly, they prefer agents that intervene or punish the offending agent, expect others to prefer them as well, and will reward the defenders as opposed to a bystander that does nothing (Kanakogi et al., 2017; Geraci, 2020; Geraci and Franchin, 2021). By 16 months of age, infants will reward a fair agent who distributes resources equally more often than they will punish this agent, and will reward the fair agent more than an unfair agent (Ziv et al., 2021). Although the victims in these studies do not express distress, infants and toddlers seem to infer or anticipate their distress which likely motivates them either to act or to expect a particular outcome. The current study adds to this research by demonstrating that young children anticipate the distress of a child who receives less than another and use that knowledge to inform their judgment of the distribution process.

The fact that the impact of emotional evaluations on fairness judgments increased with age aligns with earlier research showing that as children grow older, they increasingly consider the emotions of victims of moral transgressions (Nunner-Winkler and Sodian, 1988; Arsenio and Kramer, 1992; Keller et al., 2003). However, whereas the happy victimizer effect describes how children rate a transgressor as less happy about their transgression, in this case, older children’s focus on the “victim” who received less drives the effect on judgments.

One limitation of this study is our inability to determine the direction of causality between Emotion ratings and fairness ratings. Although our results are consistent with the possibility that children use predicted emotions to inform their fairness judgments (i.e., reasoning that it is unfair because the child who receives less will be sad), it is also possible that children use fairness judgments to inform their emotion predictions (i.e., reasoning that the victim will feel sadder because the event was unfair). However, future work should try to determine the direction of causality by directly manipulating participants’ beliefs about how recipients feel.

Lastly, our combined analysis showed that consideration of procedures and consideration of emotions independently contributed to children’s fairness judgments. Thus, children may integrate diverse kinds of information in order to form judgments of fairness, providing support for theories such as the Social Reasoning Developmental model (Elenbaas et al., 2020). Our combined results suggest that although younger children consider both procedures and outcomes when judging the fairness of resource distributions, older children increasingly integrate their concern for the welfare of the child receiving less into their judgments. Further, what changes with age is not children’s recognition that the child who receives less will be more sad, but rather the extent to which this recognition informs fairness judgments.

One potential avenue for future research is to investigate how adults integrate the same information for their fairness judgments. Generally, research on procedural justice has found that adults are more likely to accept unequal outcomes when procedures are considered fair (Lind and Tyler, 1988; Brockner and Wiesenfeld, 1996; Lind, 2019). However, several studies also show that procedures have little impact on fairness judgments when unequal outcomes are made salient (Van den Bos et al., 1997, 1998). Adults also do not accept seemingly fair procedures in all cases. For example, multiple studies in community health have found that adults reject random allocation procedures for scarce medical resources (Biddison et al., 2018; Schoch-Spana et al., 2020). Thus, when faced with large inequalities in outcomes, adults may consider many procedures inappropriate and rate the allocation process as unfair. Adults also feel empathic anger on behalf of those who are unfairly disadvantaged (Batson et al., 2007). If adults consider both the size of the inequality and expect a negative emotional impact on the disadvantaged party, they might judge the process for creating the inequality as unfair, just as the oldest children in the current study do.

## Conclusion

In sum, while a wide literature suggests that children view equal resource distributions as more fair than unequal ones (Smith et al., 2013; Blake et al., 2014; Rakoczy et al., 2016; McAuliffe et al., 2017), this study highlights that children’s judgments of inequalities change with age by integrating information about procedures, outcomes and the emotions of the recipients. Overall, with age children place greater weight on the emotions of the recipients and less weight on fair procedures used to create the unequal outcome. These findings run counter to claims that children view inequalities as fair as long as a fair procedure was used (Shaw and Olson, 2014). Instead, children’s fairness judgments are more impacted by a concern for how unequal outcomes will create distress for the disadvantaged. Future research will need to establish a stronger causal connection between emotion evaluations and fairness judgments, and perhaps test addition procedures that children may accept as more appropriate for creating large disparities in outcomes.

## Data Availability Statement

All data and code used for the analyses and figures are included as Supplementary Material.

## Ethics Statement

This research was approved by Boston University’s Institutional Review Board (#3981E). Written informed consent was provided by each child’s parent or guardian.

## Author Contributions

PB conceived of the research, analyzed the data, and co-wrote the manuscript. LS collected the data and wrote a first draft of the study. RP-L analyzed the data and co-wrote the manuscript. All authors contributed to the article and approved the submitted version.

## Conflict of Interest

The authors declare that the research was conducted in the absence of any commercial or financial relationships that could be construed as a potential conflict of interest.

## Publisher’s Note

All claims expressed in this article are solely those of the authors and do not necessarily represent those of their affiliated organizations, or those of the publisher, the editors and the reviewers. Any product that may be evaluated in this article, or claim that may be made by its manufacturer, is not guaranteed or endorsed by the publisher.
